# Scale-up and cGMP Manufacturing of Next-Generation Vaccine Adjuvant Saponin/MPLA NanoParticles (SMNP)

**DOI:** 10.1016/j.xphs.2025.103913

**Published:** 2025-07-19

**Authors:** Sammaiah Pallerla, Ivan S. Pires, Mariane B. Melo, DongSoo Yun, Andreas Wagner, Magdolna Budai, Daniel Kumar, Dietmar Katinger, Eddy Sayeed, Angela Lombardo, Darrell J. Irvine

**Affiliations:** a IAVI, 125 Broad Street, 9th Floor, New York, NY 10004; b Dept. of Chemical Engineering, Massachusetts Institute of Technology, 500 Main St., Cambridge, MA 02139; c Koch Institute for Integrative Cancer Research, Massachusetts Institute of Technology, 500 Main St., Cambridge, MA 02139; d Dept. of Immunology & Microbiology, The Scripps Research Institute, 10550 North Torrey Pines Road, IMM-312, La Jolla, CA 92037; e Polymun ScientificImmunbiologische Forschung GmbH, Donaustr. 99, 3400 Klosterneuburg, Austria; f Howard Hughes Medical Institute, 4000 Jones Bridge Rd., Chevy Chase, MD 20815

**Keywords:** Adjuvants, Saponin, SMNP, Monophosphoryl lipid A, Tangential flow filtration (TFF), Scale-up, cGMP

## Abstract

Saponin/MPLA Nanoparticles (SMNP) is a novel vaccine adjuvant that exhibited excellent safety and potency in a range of preclinical models. Successful scale-up manufacturing under current Good Manufacturing Practices (cGMP) is vital for advancing the clinical development of this promising new adjuvant. Here we report studies transitioning from small-scale formulation to the production of clinical trial material (CTM) in accordance with cGMP. By optimizing the process, a 100-fold scale increase was achieved through closed-system dilution and diafiltration, ensuring both sterility and process efficiency. Analytical characterization confirmed that the SMNP produced under cGMP conditions maintained consistent particle size, morphology, and polydispersity compared to preclinical batches. Hemolysis testing validated safety by assessing QS-21-related activity. Stability studies, conducted in accordance with ICH (International Council for Harmonisation) guidelines, demonstrated both chemical and colloidal integrity during prolonged refrigeration, while also identifying potential degradation risks at frozen or elevated temperatures. This research emphasizes critical factors for ensuring reproducibility, managing raw material variability, and developing scalable, aseptic processes. These results provide a foundation for advancing SMNP-based adjuvants into early-phase clinical trials and subsequent commercial production.

## 1. Introduction

The development of effective vaccines relies not only on potent antigens but also on adjuvants that enhance and shape the immune response [1,2]. Among next-generation adjuvants, combinations of saponins (e.g., QS-21) and monophosphoryl lipid A (MPLA) have demonstrated synergistic immunostimulatory properties, offering significant advantages over traditional adjuvant systems such as aluminum salts [3,4,5]. These advanced formulations boost both humoral and cellular responses, which are crucial for protection against complex pathogens and emerging infectious diseases [6,7]. Examples of clinical saponin/MPLA combination adjuvants include the US military vaccine adjuvant ALF-Q and the AS01 adjuvant developed by GlaxoSmithKline GSK, used in the approved Shingrix^®^, Arexvy^®^, and Mosquirix^®^ vaccines. Recently, we developed a novel adjuvant based on the self-assembly of saponin, MPLA, and phospholipids to create nanoparticles with characteristic immune stimulating complex (ISCOM)-like morphology, termed SMNP (for saponin/MPLA nanoparticles) [8]. SMNP has shown safety and exceptional potency in small- and large-animal models with a variety of immunogens, undergoing extensive testing with candidate HIV vaccine antigens [9,10,11,12,13,14,15].

Despite the promising immunological profiles of this adjuvant, transitioning from laboratory-scale formulation to GMP-compliant, industrial-scale manufacturing presents significant challenges. Saponin- and MPLA-containing formulations, like AS01 and AS02 analogs, are structurally complex and sensitive to physicochemical and process variations [16,17]. Issues such as formulation stability, raw material consistency, and in-process control become crucial as batch sizes increase [18,19]. Furthermore, the regulatory and biosafety requirements for new adjuvants necessitate thorough optimization and validation of every unit operation in the production process [19,20].

Historically, scaling up oil-in-water emulsions and liposomal systems has required iterative process development, often involving technology transfer and specialized PAT (process analytical technology) integration [21, 22]. This complexity is further compounded in adjuvants that combine multiple immunostimulants-each with unique solubility, degradation, and stabilization profiles. While various reports have detailed the successful manufacturing of aluminum- and squalene-based adjuvants [20,23,24], fewer studies address the integrated scale-up and GMP production of saponin/MPLA formulations, particularly those intended for global access and pandemic preparedness [3,25]. Recent advances in emulsion science and lipid self-assembly have improved the feasibility of scaling these systems [25]. Yet, across the industry, there remains a pressing need for reproducible, scalable, and validated processes for next-generation adjuvants that meet both performance and manufacturability standards [4,7].

In this study, we leverage a scalable laboratory method developed previously for SMNP production [26] and present a comprehensive account of the successful scale-up and GMP manufacturing of SMNP, a dual-component adjuvant system combining saponin and MPLA. We detail the transition from R&D formulations to pilot-scale and GMP-scale production, highlight the critical process parameters (CPPs) and quality attributes (CQAs) identified during development, and describe the quality control strategy that has enabled regulatory readiness. This work contributes to the field’s growing need for transparent, scalable, and globally deployable adjuvant technologies, with implications for pandemic preparedness, immuno-oncology, and emerging infectious disease vaccines.

## 2. Materials and Methods

### Materials

Cholesterol was purchased from Wilshire technologies. 1,2-Dipalmitoyl-*sn*-Glycero-3-Phosphocholine (DPPC) and synthetic MPLA (Phosphorylated HexaAcyl Disaccharide, PHAD) were procured from Avanti Polar Lipids. MEGA-10, from Bachem, and the Adjuvant QS-21 was supplied by Desert King International, USA and purchased from Indena, Italy. Chemical structures of these reagents are shown in Supplementary Table 1.

Tangential Flow Filtration (TFF) - The TFF utilized hollow fibers from Repligen equipped with a modified polyethersulfone (mPES) membrane, featuring a molecular weight cut-off of 100 kD. The filtration was operated with transmembrane pressures (TMPs) ≤ 620 mbar and shear rates ≤ 3,000/s at a QS-21 load of ≤ 0.43 mg/cm^2^.

### Identification and Quantification of QS-21

The identity and content of QS-21 were determined using reversed-phase high-performance liquid chromatography (RP-HPLC) with gradient elution and detection by a Corona Veo RS charged aerosol detector (CAD) from Thermo Scientific. During the analysis, the applied solvent gradient disrupts the nanoparticles in the sample after injection, which enables effective separation of the QS-21 components.

Chromatographic separation was performed using a Hypersil GOLD C4 column (5 μm, 4.6 × 250 mm; Thermo Fisher Scientific). The mobile phase consisted of solvent A (0.1% trifluoroacetic acid [TFA] in water) and solvent B (0.1% TFA in acetonitrile). QS-21 content was quantified by summing the peak areas of the main QS-21 compound and its QS-21b isomer. This calculation was performed using Chromeleon HPLC software (Thermo Fisher Scientific). For positive identification, the retention time of the QS-21 main peak in the sample must fall within ±0.5 minutes of the retention time of the main peak in the QS-21 reference standard.

The following linear gradient is used for elution:

**Table:** 

Minute	% solvent A	% solvent B

0.0	65	35
16.0	45	55
16.1	0	100
19.0	0	100
19.1	65	35
25.0	65	35

Analyses are performed with the following method parameters:

- Injection volume: 20 μL
- Flow rate: 1.0 mL/minute
- Column temperature: 30 °C
- Detection: CAD
  - Nebulizer temperature: 50 °C
  - Power function: 1.00
  - Filter: 3.6 s

### Identification and Quantification of MPLA, DPPC, and Cholesterol

The lipid components monophosphoryl lipid A (MPLA; congeners A and B), 1,2-dipalmitoyl-sn-glycero-3-phosphocholine (DPPC), and cholesterol were quantified using reversed-phase high-performance liquid chromatography (RP-HPLC) with gradient elution and detection by a charged aerosol detector (CAD). Prior to injection, nanoparticles were disrupted through suitable dilution in organic solvents to ensure complete lipid solubilization. Each lipid was quantified using individual calibration curves generated from a series of known standards. Peak areas were plotted against their corresponding concentrations, and quadratic regression models were used to construct the calibration curves. Quantification was conducted using Chromeleon HPLC software (Thermo Fisher Scientific), which automatically calculated lipid concentrations based on the respective calibration equations. For MPLA, the results signify the sum of congeners A and B. Positive identification of each lipid was confirmed by comparing the retention times in the sample to those of a control standard, with an acceptable deviation set at ±0.2 minutes. To assess analytical performance, four replicate injections of the control standard were performed. The measured lipid contents were compared to theoretical values to determine recovery rates. Additionally, relative standard deviations (RSDs) of both peak areas and retention times were calculated for MPLA, DPPC, and cholesterol to evaluate the precision of the method.

For analysis, the sample was diluted to a concentration in the middle of the calibrated range in 70 % isopropanol, 20 % water, and 10 % chloroform. Samples were prepared as independent triplicates. Analysis was performed using a Kinetex C18, 100 × 3 mm, 2.6 μm column (Phenomenex). Water/methanol/triethylamine/acetic acid in a ratio of 8/91/0.5/0.5 was used as solvent A and methanol/isopropanol/triethylamine/acetic acid in a ratio of 10/89/0.5/0.5 was used as solvent B. The control standard was injected three times before and once after samples were analysed. The calibration curve was generated from calibration standards with known lipid concentrations.

The following linear gradient is used for elution:

**Table:** 

Minute	% solvent A	% solvent B

0.00	100	0
5.10	77	23
5.80	75	25
7.00	75	25
10.90	10	90
11.90	10	90
11.91	100	0
15.00	100	0

Analyses were performed with the following method parameters:

- Injection volume: 6 μL
- Flow rate: 0.7 mL/minute
- Column temperature: 50 °C
- Detection: CAD
  - Nebulizer temperature: 50 °C
  - Power function: 1.00
  - Filter: 3.6 s

### Particle Size and Polydispersity Index (PdI)

Nanoparticle size and polydispersity index (PdI) were measured using photon correlation spectroscopy, also known as dynamic light scattering (DLS). This technique is based on the principle that the velocity of Brownian motion is inversely related to particle size, given known solvent viscosity and temperature. The hydrodynamic radius of the particles was calculated from the measured diffusion coefficients using the Stokes-Einstein equation. PdI served as an indicator of the width of the particle size distribution within the sample.

For analysis, the sample was diluted 1:10 in 0.2 μm filtered water; 1 mL of sample was transferred to a disposable plastic cuvette and analysed with the Zetasizer light scattering instrument (Malvern Panalytical). The sample was analysed with a refractive index of 1.450 and an absorption of 0.001, with a refractive index of 1.330 for the diluent (water). Five consecutive measurements were performed.

### Residual MEGA-10 Content

Residual MEGA-10 detergent levels were quantified using reversed-phase high-performance liquid chromatography (RP-HPLC) with UV detection at 210 nm. Separation was achieved through gradient elution, which facilitated the identification and quantification of MEGA-10 in the formulation. Analysis was performed using a Jupiter C4, 5 μm, 4.6 × 250 mm column (Phenomenex). The mobile phase consisted of 0.15 % TFA in water used as solvent A and 0.15 % TFA in acetonitrile used as solvent B. The control standard was injected 3 times before and once after analysis of the sample.

The following linear gradient was used for elution:

**Table:** 

Minute	% solvent A	% solvent B

0.0	70	30
2.0	70	30
25.0	60	40
25.1	10	90
34.0	10	90
34.1	70	30
40.0	70	30

Analyses were performed with the following method parameters:

- Injection volume: 100 μL
- Flow rate: 1.0 mL/minute
- Column temperature: 35 °C
- Detection (UV): 210 nm

### Hemolysis Assay

The hemolytic activity of the SMNP formulation was assessed using an *in vitro* hemolysis assay. Chicken erythrocytes preserved in Alsever’s solution were centrifuged to remove free hemoglobin, and the intact cells were resuspended in buffer. The erythrocyte suspension was then dispensed into a 96-well plate. Test samples were prepared by diluting the SMNP formulation in phosphate-buffered saline (PBS) and adding to erythrocytes. A QS-21 standard curve was included on the same assay plate to enable comparative analysis. Both negative and positive controls were included to validate the performance of the assay. Plates were incubated at room temperature for 30 minutes and then subjected to centrifugation. The resulting supernatant, which contained released hemoglobin, was transferred to a fresh plate, diluted 1:4, and the absorbance was measured at 412 nm (with a reference at 700 nm) using a microplate reader. The hemolytic activity of each sample was quantified relative to a free QS-21 standard curve. Results are reported as the percentage of free QS-21-equivalent hemolytic activity.

### Preparation of Precursor Detergent Solutions

The general production process for SMNP is outlined below. For each batch, lipid and adjuvant solutions were prepared individually, starting with the preparation of a 20% w/v MEGA-10 solution used for subsequent dissolution steps. The required volume of MEGA-10 solution was calculated to include both the target amount and an overage. MEGA-10 was accurately weighed and dissolved in Water for Injection (WFI) using a 37°C water bath. After complete dissolution, the solution was 0.2μm-filtered, allowed to cool to room temperature (RT), and subsequently stored at −20 ± 5°C until use.

The phospholipid DPPC and cholesterol were weighed into a pyrogen-free container and stored at – 20 ± 5°C. On the production day, these lipids were equilibrated to room temperature and dissolved in the pre-prepared 20% w/v MEGA-10 solution. Dissolution was carried out at 60°C using a water bath or heating cabinet, followed by cooling to 37 °C. The final target concentration for this lipid solution was 3.33 mg/mL cholesterol and 1.67 mg/mL DPPC.

Monophosphoryl lipid A (MPLA) was pre-weighed and stored at −20 ± 5°C before production. On the production day, MPLA was brought to room temperature and dissolved in a 20% w/v MEGA-10 solution to achieve a final concentration of 10 mg/mL. The solubilization process was conducted at 37 °C using either a water bath or heating cabinet. Once fully dissolved, the MPLA solution was added to the lipid mixture.

Next, the combined lipid/MPLA solution was added to pre-weighed QS-21 (which had been stored at 2 – 8 °C). The formulation was subsequently diluted with phosphate-buffered saline (PBS) pH 7.4 to achieve a final QS-21 concentration of 5 mg/mL.

### SMNP Particle Synthesis

Once the lipid/MPLA/QS-21 solution was fully prepared, the SMNP particle self-assembly was initiated. This was accomplished by stepwise dilution of the precursor detergent solution with PBS pH 7.4 in 11 defined stages, utilizing specific volumes and controlled flow rates to allow the saponin/lipids to equilibrate at each stage and achieve uniform and controlled particle assembly. These addition parameters were adjusted accordingly for scale-up batches. Detailed hold times and dilution volumes for each step are provided in Table 1.

Finally, ultrafiltration/diafiltration (UDF) using a hollow fiber membrane (MWCO: 100 kDa) and a tangential flow filtration apparatus was performed to remove the MEGA-10 solution from the product, exchange the buffer, and concentrate the sample to the target concentration. During ultrafiltration (UF), the sample was concentrated by a factor of 20 to minimize the diafiltration (DF) duration and buffer demand. During DF, ten volume exchanges with PBS buffer pH 6.5 were conducted. In the development study, the optimal QS-21 load was further refined, varying between different batches to identify the optimal QS-21 load per hollow fiber surface area. Subsequently, the final SMNP product was diluted to the targeted QS-21 concentration, if needed, and filled into 2R glass vials.

### TEM imaging of SMNP particles

For negative staining electron microscopy, 10 μL of the sample solution was applied to a 200-mesh copper grid coated with a continuous carbon film. The sample was allowed to adsorb for 90 seconds, after which excess solution was removed by gently touching the edge of the grid with a Kimwipe. Subsequently, 10 μL of 1% aqueous phosphotungstic acid was applied as the negative stain. After 30 seconds, the excess stain was removed by touching the edge of the grid with a Kimwipe, and the grid was allowed to air-dry at room temperature.

The prepared grid was then mounted onto a JEOL single-tilt holder and inserted into the JEOL 2100 FEG transmission electron microscope. Imaging was performed using the minimum dose method to prevent sample damage under the electron beam. The microscope was operated at an accelerating voltage of 200 kV, with magnifications ranging from 10,000x to 60,000x to assess particle size and distribution. Images were acquired using a Gatan 2k × 2k UltraScan CCD camera

## 3. RESULTS

**Figure 1.**
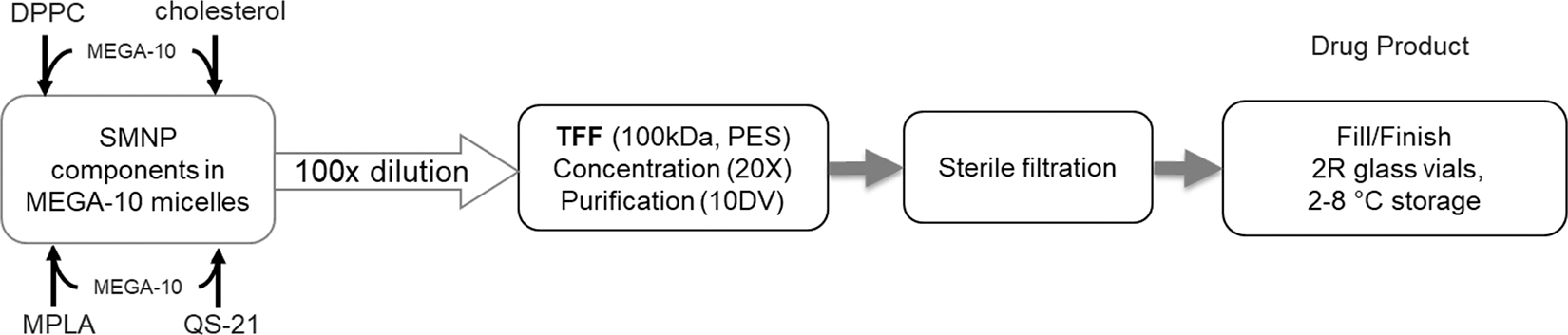
Process overview for the synthesis of SMNP.

SMNP adjuvant consists of the immunostimulators saponin and MPLA, which are self-assembled with phospholipids and cholesterol to create cage-like nanoparticles known as immune stimulating complexes (ISCOMs). Their morphology is predominantly shaped by the complexation of saponin with cholesterol and phosphatidylcholine in specific mass ratios. The formation of SMNP particles occurs by co-dissolving the lipid components in a high concentration of MEGA-10 detergent, followed removal of the detergent to facilitate particle self-assembly. At the laboratory scale, this can be easily accomplished by dialysis in buffer; however, for large-scale manufacturing, we adapted a process utilizing tangential flow filtration (TFF) that we recently described [26]. The overall cGMP manufacturing process is illustrated in Figure 1: SMNP components dissolved in MEGA-10 undergo a series of gradual controlled dilutions, followed by concentration and 10 volumes of diafiltration into PBS pH 6.5, sterile filtration, and fill/finish dispensing into 2R glass vials. The final process parameters were achieved through a series of tech transfer and scale-up manufacturing runs.

### 3.1. Tech Transfer - 15 mg scale

The initial step in the technology transfer process involved the conversion of the protocol from an academic setting to a commercial manufacturing environment. The primary objective of this first batch was to replicate the laboratory process previously established [26]. A QS-21 input of 15 mg was selected to ensure manageable processing volumes, yielding approximately 300 mL of product following dilution and about 15 mL after tangential flow filtration (TFF). These volumes were chosen as they were similar to those previously done in the academic setting.

To enhance process control and reproducibility during the 100-fold dilution step required for SMNP formation, a dosing pump was implemented. This replaced the original manual rapid dilution method used for lab-scale production, providing a more consistent buffer addition rate of approximately 250 mL/min. Particles obtained from this run were analyzed by TEM and dynamic light scattering (DLS) and showed the expected cage-like spherical morphology of SMNP (data not shown), with Z-average particle diameters of 52.1 nm and a polydispersity index of 0.137, all comparable to lab-scale preparations of SMNP (Table 2, batch SMNP/280422). Based on the findings from this small-scale run, we established quality specifications for the technology transfer batches, as shown in Table 3.

### 3.2. Scale-up - 50 mg scale

**Figure 2.**
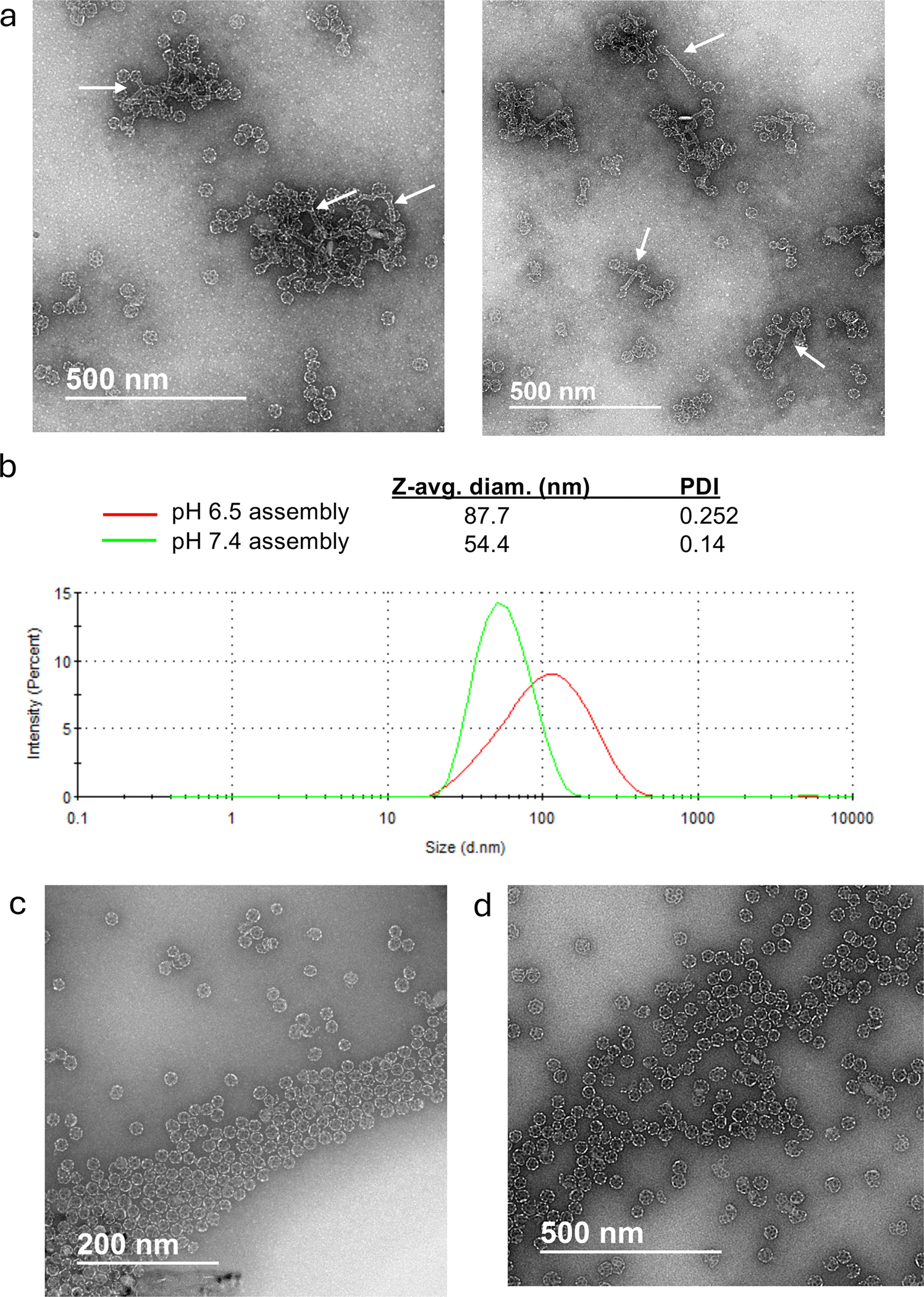
Particle morphology and size analysis from scale-up manufacturing runs. (a) TEM images of SMNP particles obtained from 50 mg scale-up batch SMNP/050522, illustrating “dumbbell” morphologies obtained when detergent dilution was carried out with pH 6.5 buffer (white arrows). (b) DLS particle size distributions obtained for adjuvant batches SMNP/050522 (pH 6.5) and SMNP/190522 (pH 7.4). (c) TEM image of adjuvant batch SMNP/190522. (d) TEM image of cGMP adjuvant batch SMNP1122-A.

For the second production run (SMNP/050522), a higher QS-21 input (4x scale-up) of 50 mg was selected to evaluate and optimize the scalability of the manufacturing process (Table 2). To limit the potential for MPLA or saponin hydrolysis, it was desirable that the final product should be stored in pH 6.5 PBS. To facilitate this end-product goal, we aimed to carry out assembly by dissolving the SMNP components with detergent and conducting the whole process starting from the detergent dilution steps using pH 6.5 PBS. The buffer addition during the dilution steps was performed at the same flow rate (~250 mL/min) as established in the 15 mg QS-21 production. A hollow fiber module with a membrane surface area of 115 cm^2^ was employed. This configuration corresponded to a QS-21 load of 0.43 mg/cm^2^, intentionally reduced compared to the 0.75 mg/cm^2^ load in the 15 mg batch, with the goal of shortening the overall TFF processing time. Unexpectedly, TEM analysis of particles formed by assembly in pH 6.5 PBS revealed the presence of both particles with the expected spherical ISCOM morphology and also “dumbbell”-like morphologies with a cylindrical structure connecting two spherical particles (white arrows, Figure 2a). Particle size distribution analysis by dynamic light scattering (DLS) revealed a Z-Avg. particle diameter substantially greater than the expected 45–50 nm and a high polydispersity index (PdI, Figure 2b and Table 2). Thus, this subtle shift in pH led to altered SMNP particle assembly.

Based on these findings, a second manufacturing run (SMNP/190522) was conducted with PBS pH 7.4 reintroduced as the buffer for the initial detergent dilution steps to induce proper SMNP cage-like assembly, while PBS pH 6.5 was retained solely for the diafiltration phase. All other process parameters, including dilution rates, were consistent with the previous batch (Table 2). This revised process produced well-formed particles exhibiting the expected spherical morphology, with particle sizes of approximately 50 nm and a PdI of less than 0.2 (Figure 2b–c and Table 2).

### 3.3 Process confirmation - Upscaling to 500 mg and material generation for Toxicology studies

A fourth production batch was conducted with a tenfold increase in QS-21 input, scaling up to 500 mg (SMNP/270722). For this batch, PBS buffer at pH 7.4 was used for the detergent dilution step to induce SMNP formation, while PBS pH 6.5 was retained for the diafiltration phase, consistent with the previous successful 50 mg batch. To accommodate the increased batch volume, the buffer addition flow rate during the SMNP formation step was slightly raised to approximately 300 mL/min. As shown in Table 2, the overall process parameters, particle characteristics, and yield at the 500 mg scale closely mirrored those established in the 50 mg batch, demonstrating the protocol’s robust scalability. TEM analysis of particles from this 500 mg scale batch showed particle morphologies indistinguishable from those of the successful smaller-scale 50 or 15 mg runs (data not shown).

Importantly, the material produced during this engineering run served a dual purpose: it was utilized both in GLP-compliant toxicology studies and as a reference standard for subsequent analytical and formulation development.

### 3.4 GMP Upscaling to 2 grams and Production of cGMP Clinical Trial Material

#### 3.4.1 cGMP Preparation for Clinical Trial Material Production

Before transitioning from process confirmation runs to Clinical Trial Material (CTM) production under current Good Manufacturing Practices (cGMP), a comprehensive GMP readiness evaluation was performed. This included verifying and qualifying all critical raw materials, reviewing and finalizing the master batch record (MBR), establishing in-process controls (IPCs) that align with identified critical quality attributes (CQAs), and validating essential equipment and analytical methods according to GMP guidelines. Earlier small-scale batches were used to refine process parameters and generate supportive comparability data, ensuring consistent product quality during the shift to larger scales. Environmental monitoring, personnel training on GMP protocols, and mock batch exercises were carried out to improve operational readiness. Collectively, these initiatives laid a strong foundation for a smooth transition to CTM manufacturing, guaranteeing that the scaled-up process would comply with regulatory standards for early-phase clinical development.

Following these preparations, a cGMP production run at a 2000 mg scale was carried out (SMNP1122-A). For the 100-fold dilution with PBS using the dosing pump system, the pump speed was optimized and increased to 360 mL/min to reduce overall process time. To accommodate the increased volumes, a stainless-steel vessel was integrated into the production process for the 2000 mg batch, where the diluted intermediate volume exceeds 20 L. All other process steps remained the same as the smaller-scale batches. Analysis of product SMNP1122-A showed successful production of SMNP at scale. DLS analysis indicated comparable particle size and polydispersity for the GMP material when compared to smaller-scale production runs, and TEM imaging of the product revealed the expected nano-cage particle morphology (Figure 2d and Table 4).

### 3.5 Stability Monitoring

A comprehensive stability assessment, following ICH guidelines, was conducted to evaluate the chemical and physical integrity of the material from batch SMNP1122-A adjuvant formulation under three storage conditions: intended long-term refrigerated (5 ± 3 °C), frozen (−20 ± 5 °C), and accelerated (25 ± 2 °C). Key quality attributes monitored over a 24-month period included QS-21 content, nanoparticle size (Zavg), and polydispersity index (PdI), which collectively reflect both the chemical potency and colloidal stability of the formulation.

#### 3.5.1 QS-21 Content Stability

**Figure 3.**
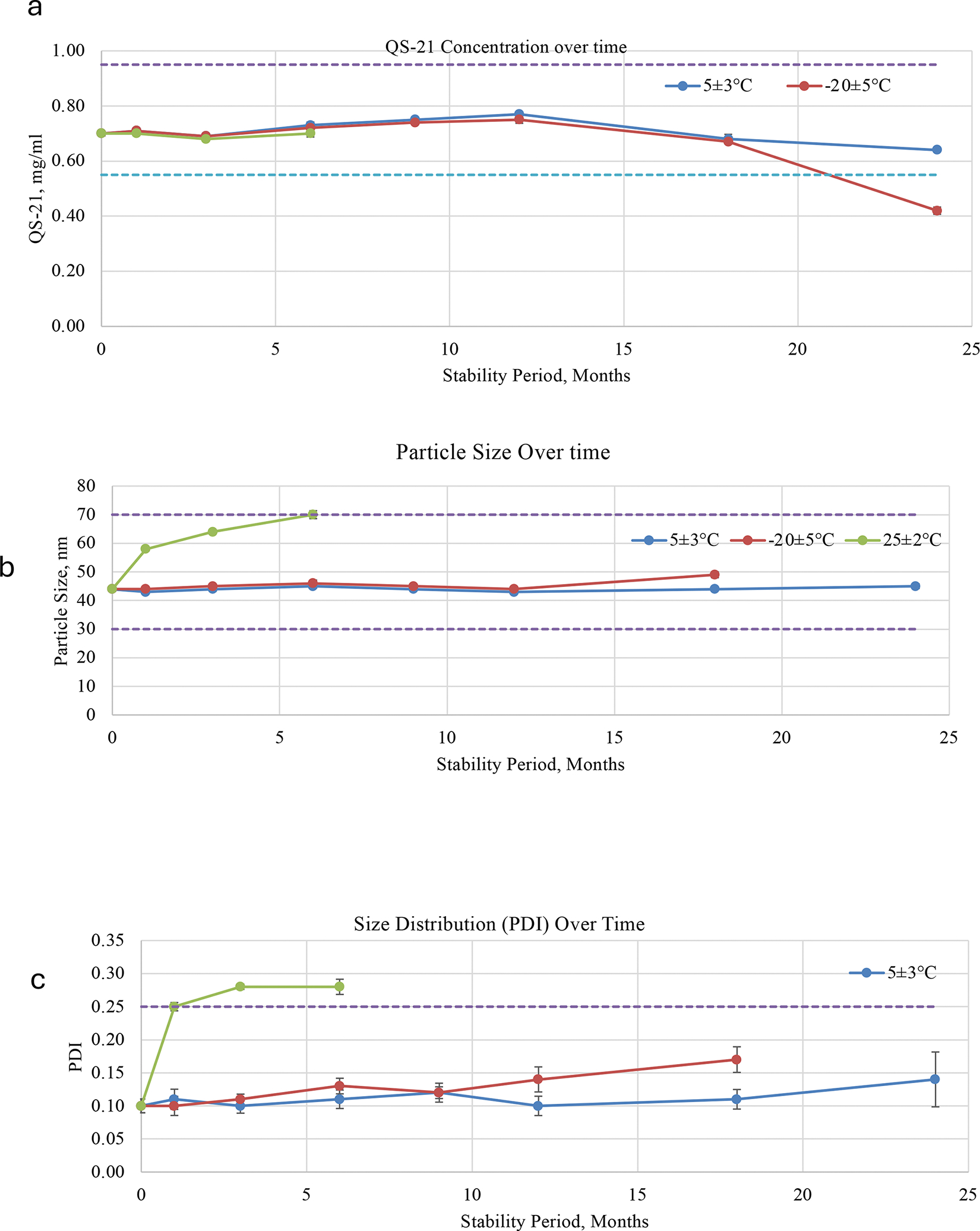
SMNP stability performance across storage conditions. SMNP vials from the cGMP production run (batch SMNP1122-A) were stored at −20°C, 4°C, or 25°C and analyzed at selected time points over 24 months. Shown are QS-21 content (**a**), Z_avg_ particle size (**b**), and polydispersity index (PdI, **c**).

QS-21 content remained stable at 5 ± 3 °C, with all values maintained within specification (0.55 – 0.95 mg/mL) for 24 months (Figure 3a). Under frozen conditions (−20 ± 5 °C), QS-21 levels remained acceptable for 18 months but declined to 0.42 mg/mL at 24 months, falling below the lower specification limit. In contrast, samples stored at 25 ± 2 °C retained stable QS-21 content for 6 months (Figure 3a); however, further time points were not evaluated due to early evidence of physical instability (see PdI section). These results suggest that QS-21 maintains chemical stability under both refrigerated and short-term room temperature conditions.

#### 3.5.2 Particle Size (Zavg) Trends

Particle size consistently remained within the target range (30 – 70 nm) across all time points under refrigerated storage, confirming physical uniformity and the integrity of nanoparticles (Figure 3b). Under frozen storage, a gradual increase in particle size was observed, with the 24-month result considered invalid due to sample dispersity, indicating aggregation or phase separation. At 25 ± 2 °C, particle size increased rapidly from 58 nm at 0M to 70 nm by 6M, reaching the upper specification limit (Figure 3b). This suggests that thermal stress at elevated temperatures leads to some degree of particle swelling, fusion, or aggregation.

#### 3.5.3 Size Distribution (PdI) Analysis

The polydispersity index (PdI) remained consistently low (≤ 0.14) and well within the specification (≤ 0.25) over 24 months at 5 ± 3 °C storage, indicating a monodisperse and stable colloidal population (Figure 3c). Under frozen conditions, the PdI progressively increased and exceeded the specification at 24 months, confirming a loss of nanoparticle uniformity. At 25 ± 2 °C, PdI values surpassed the limit as early as 3 months, suggesting an early onset of physical destabilization (Figure 3c).

#### 3.5.4 TEM Analysis

**Figure 4.**
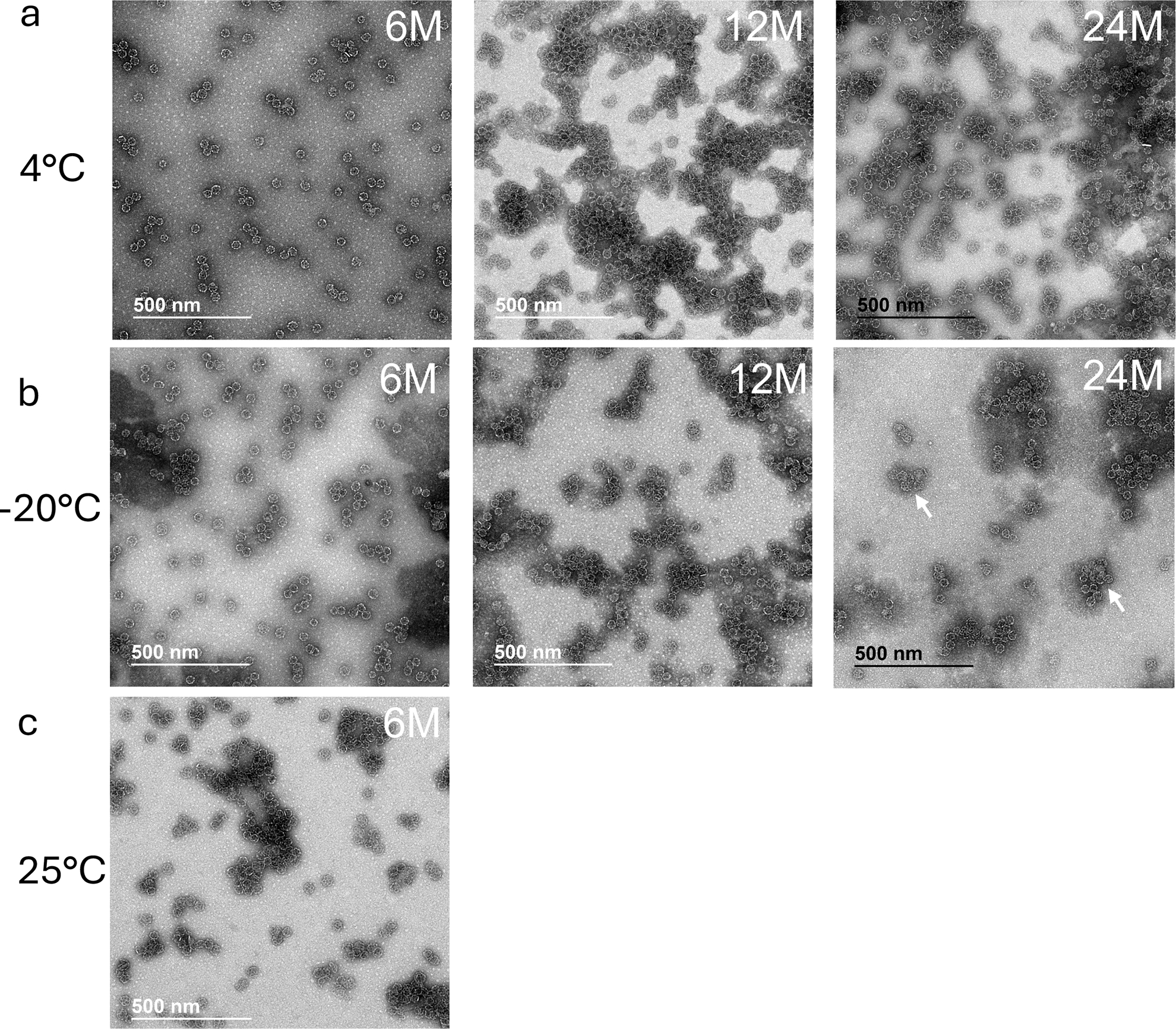
TEM analysis of SMNP under different storage conditions. SMNP vials from the cGMP production run (batch SMNP1122-A) were stored at −20°C, 4°C, or 25°C and imaged by TEM at selected time points over 24 months. Shown are images from samples stored at 4°C (**a**), −20°C (**b**), and 25°C (**c**). White arrows in (**b**) indicate potential particle fusion/aggregates.

Transmission electron microscopy imaging of the particles was conducted on samples at selected time points throughout the stability time course. SMNP stored at 5 ± 3 °C exhibited a consistent cage-like ISCOMs particle morphology over the whole 24-month storage period (Figure 4a). The adjuvant stored at −20°C did not exhibit immediately noticeable changes in particle morphology at 6 or 12 months of storage; however, some potential particle aggregation was observed in samples imaged after 24 months at this temperature (Figure 4b). SMNP stored at 25°C was only imaged at the 6-month time point and interestingly showed that the cage-like structure was maintained (Figure 4c). Overall, these analyses indicated a long shelf life of stability for SMNP stored at 5 ± 3 °C.

#### 3.5.5. Hemolytic Analysis:

Hemolytic analysis was conducted on samples collected at 6-month, 12-month, and 24-month intervals during the long-term stability study (5 ± 3 °C). This assay assesses potential changes in free QS-21 present in the preparations and was reported as the percentage of QS-21 in a quenched (non-hemolytic) state. The stability data indicated values of 92%, 93%, and 94%, respectively, demonstrating consistent hemolytic performance.

## 4. DISCUSSION

This study demonstrates the successful scale-up and cGMP manufacturing of SMNP, a next-generation nanoparticle vaccine adjuvant composed of saponin and MPLA co-assembled with lipids into ISCOM-like structures. Translating complex nanostructured adjuvants such as SMNP from research-scale protocols to clinical-grade production poses significant challenges, particularly due to their physicochemical sensitivity and the limited availability of well-defined GMP-compatible processes. Our work bridges this gap by developing and validating a robust, scalable, and aseptic production method capable of generating gram-scale quantities of SMNP with consistent physicochemical properties.

Critical to this success was the control of formulation-sensitive variables, including the buffer pH during the detergent removal step, which had a substantial impact on particle morphology and polydispersity. Reintroducing pH 7.4 PBS during detergent dilution reliably induced cage-like nanoparticle formation, while subsequent diafiltration into pH 6.5 PBS supported product stability without compromising structural integrity. These optimized conditions were preserved throughout a 100-fold scale-up and used in the final GMP batch production. The reproducibility of particle characteristics across scales was confirmed through a suite of orthogonal analytical methods including dynamic light scattering (DLS), transmission electron microscopy (TEM), and chromatographic quantification of QS-21. In-process controls and final product testing showed tight control over size, morphology, and composition, all meeting or exceeding release criteria. Notably, the process developed here eliminated the need for manual handling at critical stages, minimizing risk and enhancing scalability.

ICH-compliant stability studies demonstrated that SMNP maintains its chemical and colloidal integrity for at least 24 months at 4°C, providing a viable shelf life for clinical deployment. Degradation and aggregation were observed only under frozen or elevated temperatures, highlighting the need for cold chain storage but also revealing potential limitations associated with freeze–thaw sensitivity of the adjuvant - particularly relevant for large-scale fill-finish and distribution logistics. Aggregation observed in frozen samples appeared to arise from process-related heterogeneity during thawing rather than time-dependent degradation, issues that could potentially be addressed via cryoprotectant incorporation in future formulations. The hemolysis profile of SMNP remained stable across all time points and storage conditions tested, supporting its biocompatibility and clinical safety. These findings were further reinforced by early clinical data. Indeed, the clinical relevance of the present work is underscored by the ongoing first-in-human trial of SMNP (HVTN 144, clinicaltrials.gov: NCT06033209), in which the GMP-produced material described here is being administered in combination with a stabilized HIV Env trimer immunogen. Preliminary analyses indicate that SMNP is well tolerated and capable of eliciting potent immune responses in humans (unpublished data), validating both the adjuvant’s safety and the manufacturing platform’s clinical translatability.

The scarcity of clinical-grade adjuvants is a persistent obstacle to innovation in vaccine development, particularly for academic and nonprofit groups focused on infectious disease and global health. SMNP may help alleviate this constraint. The scalable, regulatory-aligned process described here enables the broader deployment of SMNP as a potent, adaptable adjuvant platform, opening doors to new vaccine strategies for pandemic response, immuno-oncology, and beyond.

## Conclusion

We have established a fully defined, scalable, and GMP-compliant manufacturing process for SMNP, a novel nanoparticle adjuvant combining saponin and MPLA. The process preserves key quality attributes across production scales and enables long-term refrigerated storage, providing a practical pathway for clinical translation. The successful production and clinical deployment of SMNP material-now under evaluation in an ongoing first-in-human trial-validates the formulation’s manufacturability and potential for application in humans. This work fills a critical gap in the adjuvant field and provides an immediately deployable platform for future vaccine development in both pandemic and non-pandemic contexts. SMNP stands poised to become a broadly applicable adjuvant system, empowering the next generation of high-impact vaccines.

## Supplementary Material

Supplementary Information

## Figures and Tables

**Table 1.** Dilution steps for inducing SMNP formation

Dilution step	Dilution factor	Hold times between dilution steps [min.]
1	10x	10
2	25x	45
3	30x	20
4	35x	20
5	40x	10
6	45x	10
7	50x	10
8	60x	10
9	75x	10
10	85x	10
11	100x	10

**Table 2.** Summary of the batch parameters for the small-scale experiments

Batch coding	SMNP/280422	SMNP/050522	SMNP/190522	SMNP/270722
QS-21 input, (mg)	15	50	50	500
Volume after 100-fold dilution, (mL)	300	1000	1000	10000
Dosing pump flow rate, (mL/min)	250	250	250	300
Formation buffer	PBS pH 7.4	PBS pH 6.5	PBS pH 7.4	PBS pH 7.4
Diafiltration buffer	PBS pH 6.5	PBS pH 6.5	PBS pH 6.5	PBS pH 6.5
Hollow fiber drug loading (mg/cm^2^)	0.75	0.43	0.43	0.31
Overall TFF duration, (hh:mm)	08:21	02:50	03:22	05:19
Particle size,(nm)	52.1	86.5	46.3	49.3
Particle polydispersity index	0.137	0.253	0.114	0.138
Final QS-21 concentration, (μg/mL)	739	836	760	806
Final batch volume	12.72	46.20	45.15	454
QS-21 recovery, (%)	63	77	69	73

**Table 3.** Key SMNP adjuvant Attributes

Test / Analysis	Acceptance criterion
Particle size (Zavg)	30 – 70 nm
Polydispersity index (PdI)	≤ 0.25
Residual MEGA-10	≤ 50 ppm

**Table 4:** Process parameters of 2-gram scale-up cGMP production

Test	Acceptance criterion	Result Batch SMNP1122-A
Appearance	colourless to whitish, clear to slightly opalescent suspension	whitish, opalescent
Identity QS-21	RT time conforms to reference	pass
Content QS-21	0.55 – 0.95 mg/mL	0.70 mg/mL
Identity MPLA	RT conforms to reference	pass
Content MPLA	report result	102 μg/mL
Identity DPPC	RT conforms to reference	pass
Content DPPC	report result	88 μg/mL
Identity cholesterol	RT conforms to reference	pass
Content cholesterol	report result	174 μg/mL
Particle size	30 – 70 nm	44 nm
Polydispersity index	≤ 0.25	0.10
Residual MEGA-10	≤ 50 ppm	≤ 50 ppm
pH	6.0 – 7.0	6.5
Osmolality	230 – 330 mOsmol/kg	266 mOsmol/kg
Endotoxin	≤ 50 EU/mL	< 2 EU/mL
Sterility	no microbial growth	no growth
QS-21 recovery (%)	FIO	68%

## Highlights

- Established a cGMP-compliant aseptic manufacturing process for next-generation saponin/MPLA nanoparticles (SMNP), facilitating readiness for IND-enabling studies.
- Employed orthogonal analytical methods, including CAD-HPLC for QS-21 and lipid quantification, DLS for particle sizing, and TEM for morphological validation, to confirm product integrity at scale.
- Verified SMNP safety through in vitro hemolysis assay benchmarking against QS-21 standards, ensuring acceptable biocompatibility for clinical use.
- Conducted stability studies compliant with ICH under refrigerated, frozen, and accelerated conditions, demonstrating formulation robustness and identifying degradation pathways.
