# Supplementary material for: Scale-up and cGMP Manufacturing of Next-Generation Vaccine Adjuvant Saponin/MPLA NanoParticles (SMNP)
Source: J Pharm Sci. Author manuscript; Available in PMC 2025 Aug 29. (PMC12352820; doi:10.1016/j.xphs.2025.103913)
Supplement: Supplementary Information [file NIHMS2100575-supplement-Supplementary_Information.docx]

**Supplement**

Table 1: Chemical Names, structures and molecular weights of the chemical compounds

| Chemical compound | Chemical Name, Structure and Molecular Weight |
| --- | --- |
| MPLA | Monophosphoryl Lipid A (Synthetic) (PHAD®)  C_96_H_184_N_3_O_22_P  1763.47 g/mol  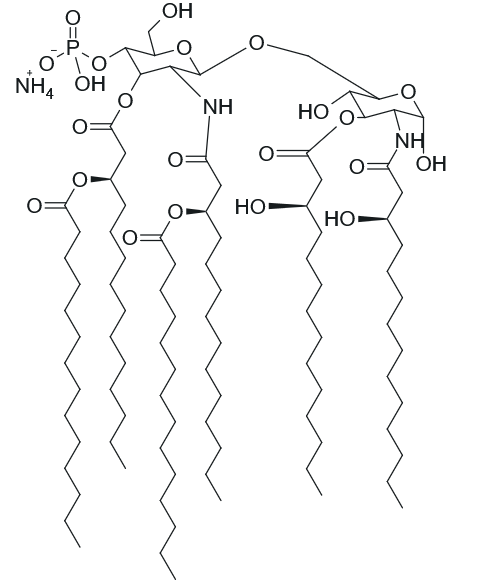 |
| QS-21 | 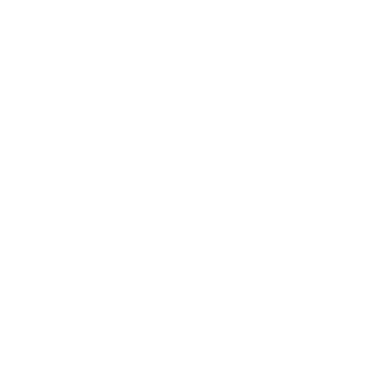C_92_H_148_O_46_  1990.13 g/mol  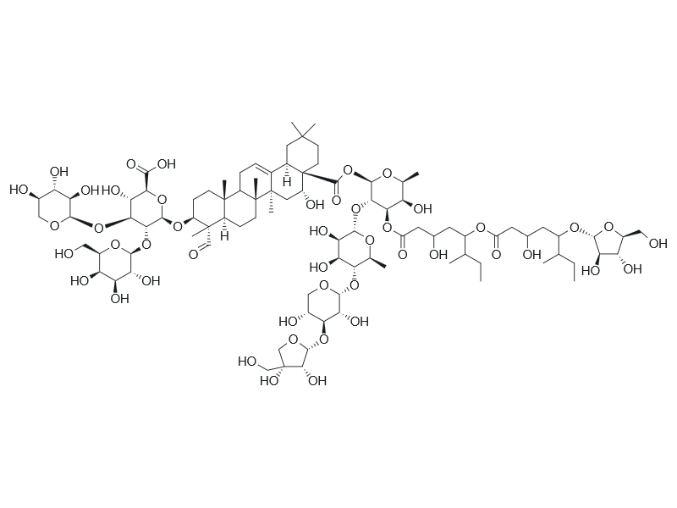 |
| DPPC | 1,2-Dipalmitoyl-*sn*-Glycero-3-Phosphocholine  C_40_H_80_NO_8_P  734.05 g/mol  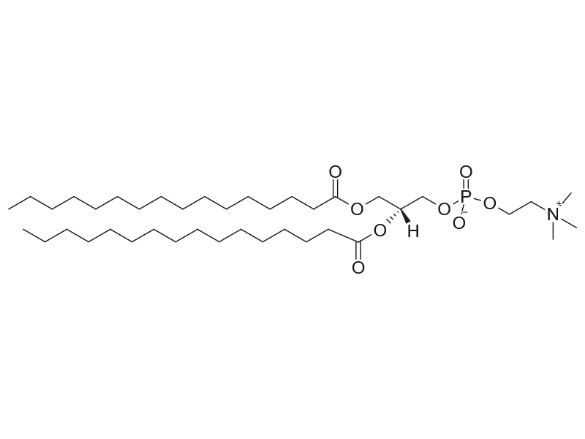 |
| Cholesterol | 5-Cholesten-3*β*-ol  C_27_H_46_O  386.66 g/mol  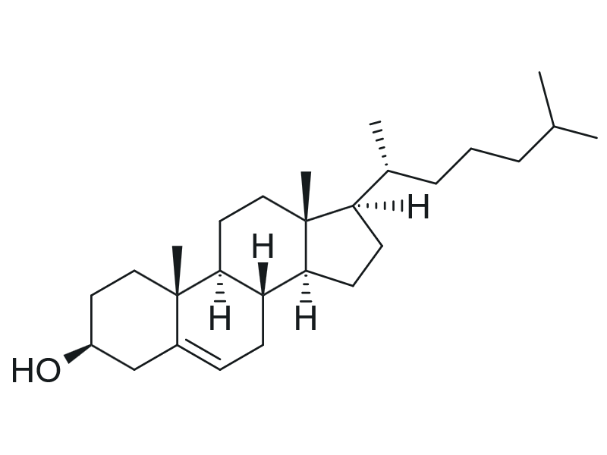 |
| MEGA-10 | N-Decanoyl-N-methyl-D-glucamine  C_17_H_35_NO_6_  349.5 g/mol  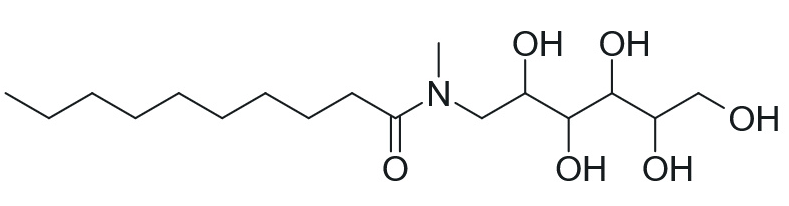 |
